# Intestinal barrier damage caused by addictive substance use disorder

**DOI:** 10.1186/s40001-025-02446-1

**Published:** 2025-04-02

**Authors:** Yan Gao, Deshenyue Kong, Jia-xue Sun, Zhong-xu Ma, Guang-qing Wang, Xing-feng Ma, Liang Sun, Hua-you Luo, Yu Xu, Kun-hua Wang

**Affiliations:** 1 Yunnan Technological Innovation Centre of Drug Addiction Medicine, Yunnan University, Kunming, 650504 China; 2 Third People’s Hospital of Kunming City, Kunming, 650041 China; 3 Drug Rehabilitation Administration of Yunnan Province, Kunming, 650032 China; 4 Department of Gastrointestinal and Hernia Surgery, First Affiliated Hospital of Kunming Medical University, Kunming, 650032 China

**Keywords:** Addictive substance use disorder, Intestinal barrier, Gut flora, The gut–brain axis

## Abstract

Addictive substance use disorder has a wide range of effects on the intestinal barrier, including damage to the biological, chemical, mechanical, and immune barriers. Damage to the intestinal barrier caused by addictive substance use disorder allows harmful substances and bacteria to cross the intestinal barrier into the circulatory system, leading to systemic inflammatory responses and immune imbalances. In addition, the interaction between the gut flora and the central nervous system is recognized as an important component of the gut–brain axis. Gut barrier damage leads to dysbiosis, which in turn affects brain function by activating immune cells and releasing inflammatory factors. This may lead to altered mood and cognitive function, increased addictive substance cravings, and dependence. Recent research has indicated that reshaping the gut–brain axis and adjusting the composition and abundance of gut microbiota holds promise in alleviating withdrawal symptoms with addictive substance dependence. This article reviews the effects of addictive substance use disorder on the intestinal barrier and explores the possibility of improving addictive substance dependence by treating gut barrier damage.

## Significant intestinal barrier damage in addictive substance use disorder patients

### Epidemiology of addictive substance use disorder

Addictive substance use disorder contributes to serious social health problems and the global burden of disease. According to *the World Drug Report 2024*, the number of individuals using addictive substances worldwide rose to 292 million in 2022, marking a 20% increase from a decade ago. Cannabis remains the most widely used addictive substance globally, with 228 million individuals reporting its use, followed by opioids (60 million), amphetamines (30 million), cocaine (23 million), and ecstasy (20 million). Globally, 64 million people suffer from addictive substance use disorders, but only 1 in 11 people receive treatment [1]. Addictive substance dependence causes significant damage to all systems of the body, and severe withdrawal symptoms often occur during the withdrawal process after addictive substance dependence. Withdrawal reactions encompass physical and psychological symptoms triggered by the withdrawal of addictive substances, such as irritability, insomnia, intense cravings, diarrhea, musculoskeletal pain, and tremors. The acute phase of withdrawal is generally recognized to last 2 weeks [2, 3], though some individuals may experience prolonged symptoms for months or years [4]. Withdrawal symptoms typically appear 8–12 h after the last use of a substance, peak between 48 and 72 h, and persist for approximately 7–14 days. Long-term use of addictive substances leads to cellular and molecular changes in the brains of individuals with substance use disorders, resulting in neuroadaptation to sustained substance use. This is referred to as neuroadaptation. Once withdrawal occurs, this equilibrium in the brain is disrupted. When cues or contexts associated with withdrawal reappear, they can reactivate memories of addictive substance withdrawal, thereby prompting individuals in the withdrawal phase to engage in compulsive addictive substance-seeking behaviors [5]. However, cravings for addictive substances in individuals with substance use disorders often intensify over time rather than diminish during prolonged withdrawal. This is a significant reason for the high relapse rate and represents the greatest challenge in current addictive substance withdrawal research [6].

### Evidence related to intestinal barrier damage in addictive substance use disorder patients

The intestinal barrier is a bridge between the external and internal environments. The gut not only maintains the balance of the intestinal internal environment, but also serves as an innate barrier to pathogenic bacteria and toxins. Functionally, the intestinal barrier can be classified as a biological barrier, a mechanical barrier, an immune barrier, and a chemical barrier [7]. Functional defects due to intestinal barrier damage are closely related to gastrointestinal diseases. A growing number of scientific studies have shown that increased intestinal permeability plays a pathogenic role in a variety of diseases, such as inflammatory bowel disease (IBD) and celiac disease, as well as functional bowel disorders such as irritable bowel syndrome (IBS) [8].

Intestinal dysfunction is also one of the serious side effects of long-term use of traditional addictive substances, manifesting itself in non-specific abdominal pain, constipation or diarrhea, malnutrition, and, in severe cases, even serious complications such as gastrointestinal bleeding and intestinal necrosis. However, compared with the systematic research on the repair mechanisms of the intestinal barrier in classic intestinal diseases such as IBD and IBS, the exploration of the molecular mechanisms of addictive substance-related intestinal barrier damage is still lacking, which restricts the development of targeted therapeutic strategies. Given that individuals with addictive substance dependence also exhibit pathological features similar to those of the aforementioned intestinal diseases, we have reason to speculate that repairing intestinal barrier damage may provide a new breakthrough direction for resolving the therapeutic bottleneck of drug dependence. Studies have shown that opioids act on the enteric nervous system, leading to dysmotility, reduced mucus secretion, and sphincter dysfunction, which in turn leads to opioid-induced bowel dysfunction (OIBD), which manifests itself in nausea, vomiting, gastroesophageal reflux, and constipation, among other related symptoms [9]. Methamphetamine (METH) is a widely used and highly addictive potent CNS stimulant, and in addition to neurotoxicity, METH causes severe intestinal barrier dysfunction [10]. Previous studies have found that METH use disorder can induce acute transient cerebral ischemic colitis, intestinal infarction, and paralytic intestinal obstruction [11–13]. According to Zhao et al. [14], METH causes intestinal inflammation by requiring the overexpression of the NLRP3 inflammasome. In METH-treated mice, the activation of the TLR4/MyD88 pathway, overexpression of protein S100A8/A9, and increased levels of serum pro-inflammatory cytokines lead to increased intestinal permeability [15]. Heroin and morphine are representative opioid alkaloids, which exert their pharmacological effects by binding to opioid receptors (OR). ORs are widely and differentially expressed in both the central and peripheral nervous systems. Upon activation, ORs can inhibit adenylate cyclase, activate K^+^ channels leading to hyperpolarization of enteric neurons, and close Ca2^+^ channels to inhibit the release of neurotransmitters. This results in reduced excitability of intestinal muscles and submucosal neurons, leading to decreased intestinal motility and reduced mucus secretion from the intestinal mucosa [16, 17]. In addition, morphine mediates intestinal injury by regulating the production of nitric oxide (NO), while impairing the functional state of macrophages to reduce the body’s immune defense barrier function [18]. Long-term exposure to morphine not only induces intestinal inflammation in mice, but also leads to intestinal dysbiosis and increased intestinal epithelial permeability [19]. Chronic morphine treatment can alter the composition of resident gut microbiota and induce bacterial translocation across the intestinal epithelial barrier through mechanisms involving Toll-like receptors (TLR) [20]. Chronic cocaine use causes gastrointestinal disorders in humans, including symptoms like constipation or diarrhea, as well as damage to the gastrointestinal tract, affect the gut microbiota [21]. It is worth noting that prescription addictive substances can also cause intestinal inflammation. For example, addictive substances-induced enteritis often occurs after long-term and high-dose use of broad-spectrum antibiotics. Nonsteroidal anti-inflammatory drugs (NSAIDs) can cause gastrointestinal damage, induce relapse of IBD in remission, and cause colonic ulcers, erosions, and stenosis [22]. Immunosuppressants and immune checkpoint inhibitors (ICIs) can also induce the occurrence of intestinal inflammation [23]. Here, we only discuss the intestinal damage caused by addictive substance use. All four major intestinal barriers are damaged by addictive substance use disorder, and this damage not only interferes with normal intestinal function, leading to impaired nutrient absorption, but may also disrupt the tight junctions (TJs) proteins of the intestinal epithelium, increase intestinal permeability, and allow bacteria and toxins to enter the bloodstream, which can lead to systemic inflammation. More seriously, damage to the intestinal barrier may further trigger multi-organ dysfunction and even lead to death.

#### Microbial barrier damage

The intestinal *microbial* barrier is a microecosystem composed of resident flora that live in symbiosis with the organism, which are relatively constant in number and distribution, both interdependent and interactive, and are mainly composed of aerobic, anaerobic, and parthenogenetic anaerobic bacteria. Specialized anaerobes not only have antimicrobial and intestinal-immunity regulating effects, but also lower intestinal pH, and acidify the intestine by secreting short-chain fatty acids (SCFA) and lactic acid, and inhibit the growth of invasive flora by competitively ingesting enteric nutrients [24]. Intestinal homeostasis depends on gut microbiota, antigens in food, hormone levels, immune function, living environment, daily behavior patterns, and interactions between hosts. Disrupting this relationship will directly affect the immune system, barrier function, and the composition of the intestinal microbiota, disrupting intestinal homeostasis [7].

Gut flora plays an important role in the intestinal ecosystem, mainly composed of Bacteroidetes, Firmicutes, Proteobacteria, and Actinomycetes, which are involved in a variety of physiological processes, such as food digestion, immune regulation, and maintenance of the intestinal mucosal barrier. A large number of animal and human experiments have shown that the abuse of METH, cocaine, and opioids such as heroin, morphine, and methadone can cause significant changes in the diversity of the gut flora and weaken the intestinal barrier (see Table 1). First, addictive substance dependence leads to a decrease in the relative abundance of some Firmicutes, and some key species of Firmicutes, such as *Lactobacillus*, play an important role as beneficial intestinal bacteria in maintaining intestinal health [25]. However, addictive substance dependence often leads to a down-regulation of the abundance of these beneficial bacteria, thereby diminishing their protective effects on the host. In contrast to Firmicutes, addictive substance use disorder dependence often leads to an increase in the relative abundance of certain species in Bacteroidetes, whose imbalance may trigger gut inflammation and other health issues. In addition, addictive substance use disorder also has an impact on a portion of the flora in Actinobacteria and Ascomycetes. In particular, certain species may see a rise in abundance while others may see a fall. These alterations imply that addictive substance use disorder causes a significant disturbance in the variety and equilibrium of the gut flora, which impacts not only the digestive system’s regular operation but also a host of other health issues, such as immune system disorders, intestinal inflammation, and infections. In summary, addictive substance use disorder significantly alters the gut microbiota and impairs the integrity of the intestinal mucosal barrier, highlighting the role of gut microbiota in the pathophysiology of substance use disorders and withdrawal symptoms. In-depth research on the changes in gut microbiota in patients with addictive substance use disorders, and the development of targeted intervention strategies to repair intestinal barrier damage based on these findings, holds promise for improving addictive substance use disorder conditions.

**Table 1:** Changes in gut flora composition caused by addictive substance use disorder

Addictive substances	Species	Methods	Major results	References
Opioid	Human	Compare the fecal microbiota composition of cirrhotic patients who take and do not take opioids	Bacteroidaceae$\downarrow$	[91]
Opioid	Human	Analyze the fecal microbiota composition of African American males with opioid use disorder (n = 99)	*Bifidobacterium*$\uparrow$	[25]
Opioid	Human	Collect fecal samples from patients with substance use disorders who use and do not use agonists and antagonists for analysis	*Roseburia*$\downarrow$, *Bilophila*$\downarrow$	[92]
Methadone	Human	Comparison of fecal microbiota composition in non-opioid users and methadone-treated individuals	Actinobacteria$\uparrow$, Verrucomicrobia$\downarrow$	[93]
METH	Human	Select subjects with METH use disorder and healthy subjects matched for age and gender to analyze the differences in gut microbiota	Sphingomonadales$\uparrow$, Xanthomonadales$\uparrow$, *Romboutsia*$\uparrow$, Lachnospiraceae$\uparrow$; Deltaproteobacteria$\downarrow$, Bacteroidaceae$\downarrow$	[94]
Cocaine	Human	Recruit HIV-infected individuals (n = 15) and participants who are not infected with HIV, both of whom have used cocaine within the past month, to analyze gut microbiota	Bacteroidetes$\uparrow$, Firmicutes$\downarrow$	[95]
Morphine	Mouse	Treat mice with sustained-release morphine pellets or placebo pellets for 16 h to measure changes in gut microbiota	*Parasuterella excrementihominis*$\uparrow$, *Enterococcus faecalis*$\uparrow$, *Enterorhabdus caecimuris*$\uparrow$; *Lactobacillus johnsonii*$\downarrow$	[96]
Heroin	Mouse	Continuous injection of 10 mg/kg heroin for 21 days, and the feces of mice were collected for analysis	*Bifidobacterium*$\uparrow$, *Sutterella*$\uparrow$; *Akkermansia*$\downarrow$	[97]
METH	Mouse	C57/BL6 mice were injected with METH (15 mg/kg) to induce anxiety-like behavior, and the gut microbiome was analyzed	*Roseburia*$\uparrow$, *Lactobacillus*$\uparrow$, *Mucispirillum*$\uparrow$, *Rikenella*$\uparrow$, *Bifidobacterium*$\uparrow$;	[29]
Cocaine	Rat	Male rats were exposed to cocaine, caffeine, or phenacetin smoke for 14 days to analyze changes in the gut microbiome	Lachnospiraceae$\uparrow$, Prevotellaceae$\uparrow$; Spirochaetaceae$\downarrow$, Desulfovibrionaceae$\downarrow$	[98]
METH	Rat	Male rats were injected twice daily with saline for the first 14 days, followed by twice daily injections of 2 mg/ml METH for the next 14 days, and fecal samples were collected for analysis	Actinobacteria$\uparrow$, Bacteroidetes$\downarrow$	[65]

#### Mechanical barrier damage

The mechanical barrier, also known as the physical barrier, is based on the intestinal mucosal epithelial cells and the intercellular junction complex. This complex consists of three types of junctions, the apex is composed of TJs to form a selective osmotic barrier, the Adherens junction (AJs) below it maintains intercellular adhesion, and the desmosomes distributed in the lateral membrane provide mechanical resistance [26], which effectively block the entry of bacteria, viruses, and endotoxins. Damage to the intestinal mucosal barrier function is closely related to structural and functional abnormalities of TJs in intestinal epithelial cells. It has been found that alterations in the TJs between intestinal epithelial cells in patients with ulcerative colitis lead to intestinal mucosal barrier dysfunction [27].

According to recent research, addictive substance use disorder alters the physical barrier lining the intestines, increasing intestinal permeability, allowing toxins and bacteria from the gut to enter the circulation, and causing an inflammatory reaction throughout the body. It has been discovered that intestinal epithelial cells in HIV-positive individuals undergo apoptosis, and the tight junction complex is harmed [28]. Furthermore, via activating the TLR4/MyD88/NF-κB pathway, which also markedly down-regulated the expression of two TJ proteins, Claudin-5 and Occludin, METH causes hippocampal neuroinflammation [29]. Cocaine, on the other hand, controlled the expression of TJ proteins, damaged the intestinal barrier and altered permeability, which in turn caused bacterial translocation, the entry of bacterial metabolites into the bloodstream, and immunological damage. Cocaine also inhibited actin assembly and altered the conversion of F-actin/G-actin [30]. To summarize, addictive substance use disorder weakens the function of the intestinal physical barrier by damaging intestinal epithelial cells, disrupting intercellular TJs, and altering the metabolic and signaling activities of these cells (refer to Table 2). In summary, the impact of addictive substance use disorder on TJ protein expression and intestinal mechanical barrier degradation is a multifaceted problem involving a variety of processes. An in-depth understanding of these effects can help develop targeted interventions to maintain gut health and improve rehabilitation outcomes for individuals with addictive substance use disorder.

**Table 2:** Mechanical barrier damage caused by addictive substance use disorder

Addictive substances	Species	Major results	References
METH	Mouse	METH significantly down-regulates the expression of two TJ proteins, Claudin-5 and Occludin	[29]
METH	Rat	Intestinal TJ proteins claudin-1 and zonula occludens-1 are significantly reduced and permeability is increased in HIV-1 transgenic rats	[31]
Cocaine	Rat	Cocaine affects F-actin/G-actin conversion by inhibiting actin assembly	[30]

#### Immune barrier damage

The intestinal immune barrier, as an important line of immune defense for the body, is mainly composed of Gut-Associated Lymphoid Tissue (GALT) as well as Secretory Immunoglobulin A (SIgA). GALT is essential for maintaining the integrity of the intestinal mucosal barrier, not only against pathogens, but also to ensure that food antigens and commensal microorganisms remain immune-tolerant [32]. The gut microbiota maintains intestinal homeostasis through dynamic interactions with the host innate and adaptive immune system; however, addictive substance use disorder can alter the composition and function of the gut flora, leading to dysregulation of the immune system, which can cause intestinal inflammation and tissue damage [33].

In addictive substance use disorder patients, intestinal barrier damage significantly activates innate immune cells in the gut, inducing the release of inflammatory factors; bacterial translocation and the entry of bacterial metabolites into the bloodstream significantly activate peripheral immune cells, ultimately leading to immune imbalance. Long-term treatment with METH leads to a significant decrease in activated CD4^+^ and CD8^+^ T cells and other T lymphocyte lineages [34–36], a significant reduction in the proliferation rate and activity of splenic NK cells, and an increase in B cell infiltration [37–39]. Intestinal barrier damage associated with acute METH withdrawal can induce a cytokine storm. During the acute withdrawal period, patients show a significant decrease in serum IL-1β, IL-9, IL-15, FGF, and MIP1α, while IL-1Rα, IL-6, Eotaxin, IP-10, and VEGF are significantly increased. After long-term withdrawal, IL-6, IL-7remain significantly higher than the control group, concurrently, the number of peripheral blood CD3^+^ T cells and CD4^+^ T cells is significantly lower than the control group [40]. This suggests that the immune imbalance caused by METH use disorder shows significant changes during the acute phase and remains unrecovered even after long-term withdrawal; similar changes are also observed in patients undergoing heroin withdrawal [41]. Clinical studies on METH use disorder and patients with HIV infection have revealed that stimulant use may aggravate the development of clinical HIV by increasing the activation of monocytes [42]. Similarly, opioid use disorder also shows significant immunosuppressive function [43]. Ennis et al. [44] showed that a variety of opioid treatment can change the histamine secretion of mast cells, and the function of mast cells will also be inhibited. It can also reduce the interaction between macrophages, such as antigen presentation, resulting in reduced antibody production [45]. After morphine treatment, the expression of chemokines in the intestinal tissue of mice significantly increased, followed by an increase in neutrophil infiltration, a decrease in the number of macrophages, and an impact on their proliferative capacity. This leads to a significant delay in the recruitment of macrophages and neutrophils to inflammatory sites, which is a direct result of intestinal microbial dysbiosis [46–48]. In summary, both clinical and basic experiments have confirmed that substance use disorders can cause immune imbalance, damage the intestinal immune barrier, disrupt the balance of the intestinal microbiota, and promote the occurrence and development of intestinal inflammation (as shown in Table 3). The intestinal mucosal barrier plays a crucial role in the human immune system. Damage to the intestinal mucosal barrier makes it easier for harmful microorganisms and toxins to cross the mucosal barrier and enter the intestinal tissue, thereby stimulating the immune system’s response and triggering inflammation. At the same time, due to the abnormal activation and functional disorders of immune cells, the destruction of the intestinal mucosal barrier can further deteriorate, forming a vicious cycle, leading to the occurrence of immune imbalance. These immune imbalances can affect the blood–brain barrier (BBB) and the CNS, resulting in neuroinflammation and changes in brain function.

**Table 3:** Immune barrier damage caused by addictive substance use disorder

Addictive substances	Species	Major results	References
METH	Human	The increased activation of monocytes aggravates the development of clinical HIV	[42]
Morphine	Mouse	The expression of chemokines in intestinal tissue of mice increased significantly, and neutrophil infiltration increased	[46]
Fentanyl, morphine	Mouse	Damage the function of macrophages, natural killer ( NK) cells and T cells	[43]
Opioid	Mouse	The histamine secretion of mast cells is changed and the function of mast cells is inhibited	[44]
Morphine	Mouse	The decrease in the number of macrophages and the decrease in the ability to proliferate significantly delay the recruitment of macrophages and neutrophils to the inflammatory site	[47]
Fentanyl, buprenorphine	Mouse	Reduced interaction between macrophages, such as antigen presentation, resulting in reduced antibody production	[45]

#### Chemical barrier damage

The intestinal chemical barrier is composed of mucus secreted by mucosal epithelial cells, digestive juices secreted by the gastrointestinal tract, and bacteriostatic substances produced by intestinal commensal bacteria, which are bactericidal as well as prevent the invasion of pathogenic bacteria. The mucus layer is the first line of defense in the intestinal chemical barrier. It is composed of mucus secreted by intestinal epithelial cells and prevents pathogens and harmful substances from directly contacting epithelial cells [49]. The mucus layer also provides a habitat for intestinal microbes, promoting the growth and colonization of beneficial bacteria, thereby influencing the composition and function of the gut microbiota to a certain extent [50]. The intestinal mucosal epithelium is interspersed with a large number of goblet cells. The mucin (MUC) secreted by goblet cells can serve as ecological niches for bacterial adhesion, competitively binding bacteria that might otherwise attach to the intestinal epithelial cells, thereby confining bacteria within the mucus layer and expelling them from the body with the mucus during intestinal peristalsis [51]. The main intestinal antibacterial substances include bile, mucopolysaccharides, lysozyme, and glycoproteins. The highly acidic environment in the stomach, formed by gastric acid, prevents the majority of bacteria and fungi from colonizing the stomach [52]. Bile acids can influence the composition of the gut microbiota and inhibit the growth and proliferation of intestinal pathogens [53, 54]. Lysozyme can destroy the cell walls of bacteria, causing them to lyse [7]. Studies have shown that the metabolites of gut microbes (e.g., tryptophan, polyamines, and amino acids) influence human behavior and physiology to some extent [55]. For example, γ-aminobutyric acid (GABA) is a known regulatory molecule of the CNS and intestinal nervous system and is important for maintaining the normal function of the nervous system and intestines; histamine regulates the function and activity of intestinal immune cells by binding to corresponding receptors on the surface of intestinal immune cells; and SCFAs produced by the gut microbiota are important for the maintenance of normal intestinal and intestinal function by regulating immune function, promoting nutrient absorption, maintaining acid–base balance, and increasing mucus secretion to maintain intestinal homeostasis [56]. In previous studies, we collected detailed information and clinical samples from METH-dependent individuals and established a rhesus monkey model of METH dependence. The research results showed that both METH-dependent individuals and the rhesus monkey model exhibited a significant decrease in body mass index, a significant increase in serum D-lactate dehydrogenase (D-LDH) and diamine oxidase (DAO) levels, and varying degrees of intestinal mucosal epithelial erosion, villous degeneration and necrosis, and destruction of the lamina propria glands in different segments of the intestine [57]. These results were also observed in *individuals with heroin use disorder* and mouse models during the withdrawal period [58]. In summary, addictive substances significantly affect the intestinal chemical barrier. On the one hand, the imbalance of gut flora and intestinal metabolites associated with addictive substance use disorder can directly alter the intestinal environment and cause chemical barrier damage; on the other hand, the immune imbalance caused by addictive substance use disorder leads to a decrease in the synthesis of antimicrobial enzymes and other substances, which results in a significant impairment of the chemical barrier. These alterations not only weaken intestinal defenses against pathogens, but may also lead to abnormal digestive function and an imbalance in the intestinal environment.

## Intestinal barrier damage exacerbates altered brain function

**Fig. 1:**
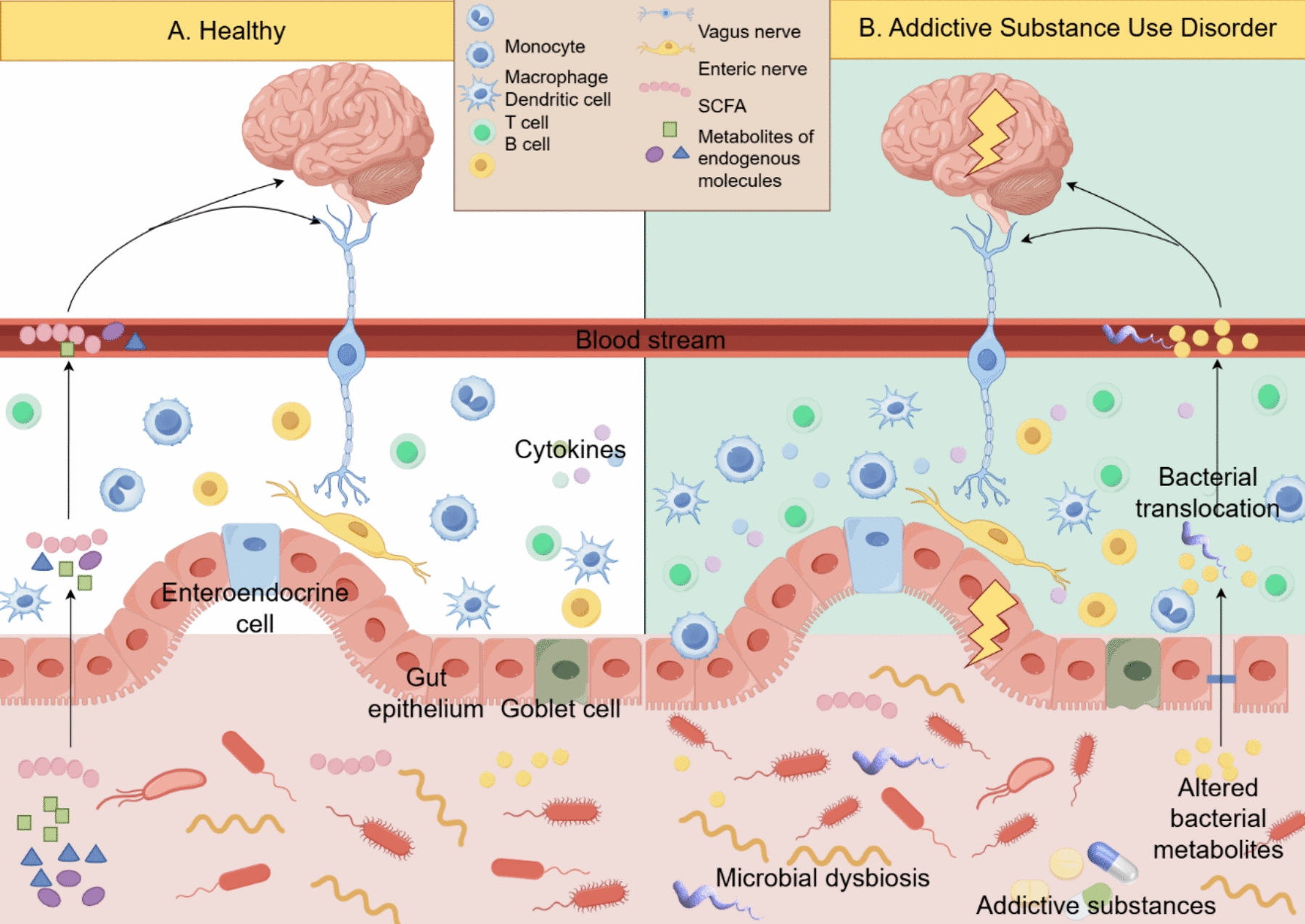
Effects of addictive substance use disorder on gut–brain axis

The effects of addictive substance use disorder on the human body are wide-ranging and far-reaching, including behavioral, biochemical, and toxic organic consequences, especially on the brain. In terms of behavioral effects, addictive substance use disorder can lead to altered cognitive function, impaired judgment, and changes in mood and emotions. These substances often disrupt the brain’s reward system, leading to addictive behaviors and compulsive addictive substance use. At the biochemical level, addictive substance use disorder can interfere with the normal function of neurotransmitters, such as dopamine, serotonin, and GABA, affecting mood regulation, reward processing, and overall brain function. In addition, chronic addictive substance use disorder can have toxic organic effects on the brain, leading to structural and functional damage. Changes in the gut flora, acting synergically with an individual’s genetic background, can alter the enteric nervous system, the CNS, and the immune system, impair barrier function, and lead to a variety of diseases [59]. Intestinal barrier damage allows leakage of gut contents such as gut flora, toxins, and inflammatory cells into the circulatory system, and gut-derived components that escape into the circulatory system can enter the CNS, cross the BBB, and trigger an inflammatory response within the brain [60]. The gut and brain are not only physically connected through the vagus nerve but also chemically connected through metabolites, hormones, and neurotransmitters [61]. The vagus nerve, a major component of the parasympathetic nervous system, mediates the cholinergic anti-inflammatory pathway, which suppresses peripheral inflammation and reduces intestinal permeability, thereby modulating gut flora composition. In turn, gut flora can also influence brain plasticity via the vagus nerve [62]. The gut–brain axis includes biochemical signaling that occurs between the gastrointestinal tract and the CNS [63]. Studies have shown that METH can affect the gut–brain axis through a variety of mechanisms, such as altering the gut flora, increasing intestinal permeability, activating immune responses or inflammation, and increasing BBB permeability [64]. The gut flora is a key component of the gut–brain axis, and disturbances in the homeostasis of this system have been associated with a variety of behavioral and neurological disorders, such as Parkinson’s disease, Alzheimer’s disease, autism, anxiety, depression, and substance use disorders [65]. Gastrointestinal dysfunction is an important factor in the pathogenesis of Parkinson’s, and the intestine may even serve as a pathway for the spread of pathology to the CNS [63]. The amount and composition of the gut flora and microbial metabolites are significantly altered in patients with Parkinson’s disease, with significant reductions in *Prevotellaceae* in fecal samples from patients with Parkinson’s disease compared to healthy individuals [66, 67]. In addition, dysbiosis of the gut flora induces lymphocyte activation, leading to systemic inflammation, as well as triggering neuroinflammation regulated by astrocytes and microglia, causing imbalance in the CNS homeostasis, and subsequently resulting in brain dysfunction and behavioral disorders [68]. Finally, gut flora alterations can also directly modulate CNS activity through the gut flora–vagus nerve. Intestinal dysbiosis and metabolic changes can lead to disruption of the internal and external ecological balance, and the vagus nerve can sense these changes and regulate the CNS [69], causing changes in brain function and behavior. Increased permeability of the intestinal barrier leads to the invasion of different bacteria, viruses, and their neuroactive products, which support the neuroinflammatory response in the brain [70]. The intestinal barrier and brain function are closely linked through the gut–brain axis, which transmits information through neural, endocrine, and immune pathways. Changes in the gut microbiota cause intestinal immune and inflammatory responses, which in turn affect brain function through the gut–brain axis (see Fig. 1).

The gut–brain axis achieves bidirectional interaction through immune, neural, and endocrine mechanisms. Gut microbiota influence the CNS via SCFAs, endogenous metabolites, and other pathways. Substance use disorders lead to an increased proportion of pro-inflammatory microbiota, reduced abundance of SCFA-producing bacteria, alterations in bacterial metabolites, and bacterial translocation into the bloodstream. These translocated components can penetrate the BBB, triggering neuroinflammatory responses in the brain. This inflammatory response, in turn, further exacerbates damage to the intestinal barrier. (By figdraw).

## Reshape the gut–brain axis to indirectly improve addictive substance use disorder

Individuals with addictive substance use disorder often suffer from gastrointestinal dysfunction and mental health issues, such as anxiety and depression. Changes in the gut microbiota are directly linked to mental disorders like depression, anxiety, and cognitive dysfunction [71]. It has been reported that the supplementation of probiotics may have a positive impact on alleviating depressive symptoms, minimizing hippocampal damage, and improving cognitive abilities [72]. Additionally, probiotics can regulate immune responses by mitigating excessive inflammatory reactions [73] and coordinating the differentiation of naïve T cells [74, 75]. They can also enhance the secretion of neuroactive metabolites and neurotransmitters, thereby contributing to the improvement of CNS disorders [76]. In recent years, fecal microbiota transplantation (FMT) have made progress in autoimmune diseases, diabetes, depression, Parkinson’s disease, and addictive substance use disorder, and intestinal microbiota and related metabolites have also become a hotspot for addictive substance target screening. FMT, a method of transplanting fecal microbiota from a healthy donor into the gastrointestinal tract of a patient, has been shown to be an effective means of treating *Clostridium difficile* infections and IBD [77]. A study found that transplantation of fecal microbiota from mice supplemented with *Clostridium butyricum* improved cognitive performance and colon integrity, suggesting that *Clostridium butyricum* influences cognitive function by remodeling gut flora [78]. Feeding deoiled and dechlorogenic sunflower seeds prevented depressive-like behavior, intestinal barrier damage, elevated plasma corticosterone, decreased hippocampal serotonin levels in mice, and significantly changed the structure of the gut flora, suggesting that depression can be effectively prevented in mice by remodeling the gut flora and bacterial tryptophan metabolism [79]. In addition, oral administration of chiral Au nanoparticles effectively ameliorated neuroinflammation and reversed Alzheimer’s disease in the mouse brain by altering the composition of the intestinal microbiome and increasing the intestinal metabolite indole-3-acetic acid, which significantly improved cognitive performance [80].

Addictive substance use disorder leads to dysbiosis of the gut microbiota, which in turn results in damage to the intestinal barrier function, increasing the production and absorption of harmful metabolic products, thereby having adverse effects on the CNS. Adjusting the composition and abundance of the gut microbiota can alleviate withdrawal symptoms and addictive substance-seeking behaviors in animal models of addictive substance use disorder. FMT as an emerging therapeutic approach, has also shown great potential in the treatment of symptoms related to addictive substance use disorder. It was shown that Gegen-Qinlian decoction (GQD) can effectively alleviate the intestinal microenvironment and anxiety-like behaviors in mice during the withdrawal period, and this effect is dependent on the remodeling of the abundance and restoration of the metabolite levels of *Akkermansia*, which exerts anxiolytic effect through the target of “*Akkermansia*–metabolite–intestinal metabolite–gut flora” [81]. In addition, similar to the aforementioned addictive substances, metformin also relies on the remodeling of gut microbes and metabolites to improve METH withdrawal symptoms [82]. These research findings suggest the potential to improve symptoms related to addictive substance dependence by reshaping the gut microbiota. However, it should be noted that the mechanisms and specific effects of the gut microbiota–brain axis in the treatment of addictive substance use disorder still require further research. Moreover, FMT is still in the research phase, and there is currently insufficient research evidence to support the therapeutic effects of FMT on symptoms related to addictive substance use disorder. In addition, the application of FMT still faces some challenges, such as donor screening, and the safety and efficacy of the transplantation process. Therefore, effectively eliminating pathogenic bacteria associated with intestinal diseases without affecting the gut microbial ecosystem remains the most formidable challenge at present.

## Summary and outlook

Up to now, the specific mechanism of addictive substance use disorder is still unclear. At present, the drugs that can be used to alleviate the symptoms of addiction withdrawal are extremely limited, and no effective and safe withdrawal methods and drugs have been found. For drug withdrawal, the most important treatment is drug substitution, such as methadone, as an opioid receptor antagonist, which is widely used to treat opioid dependence [83]. However, methadone itself has a great potential safety hazard. Long-term use of methadone may cause neurotoxicity, and may even lead to drug dependence on methadone. Death cases caused by excessive use of methadone exist at any time [84]. The key to the treatment of addictive substance use disorder is to solve the problem of withdrawal, how to alleviate the withdrawal symptoms, improve the success rate of withdrawal, and reduce the relapse rate are the problems that need to be solved urgently in the current addictive substance use disorder.

Intestinal flora and metabolic remodeling are the core mechanisms of many drugs and the targets of new interventions. Although some studies have shown that there is a complex and subtle relationship between intestinal flora and individual behavior, emotion and even response to drugs, suggesting that the adjustment of intestinal flora may become an innovative way to treat addictive substance use disorder, the research in this field still faces many challenges and unknowns, and it will take time to truly apply it to clinical practice. First of all, an in-depth understanding of the interaction mechanism between gut microbiota and addictive substance use disorder will help to develop new treatment strategies. This includes identifying the role of specific intestinal microbial communities and their metabolites in addictive substance use disorder induced intestinal damage, and exploring how to target these specific microorganisms and metabolites to achieve therapeutic effects [85, 86]. Secondly, diet is an important factor affecting intestinal flora. Changes in dietary structure can significantly affect the composition and function of intestinal flora [87]. Therefore, formulating reasonable dietary recommendations to promote the growth of beneficial bacteria is one of the important strategies for reshaping the gut microbiota. In particular, a high-fiber diet is believed to help increase the proportion of beneficial bacteria [88]. In addition, the interaction between the gut microbiota and the host is a complex network involving multiple aspects such as the immune system, metabolic pathways, and the nervous system [89]. Therefore, in-depth research on the interaction mechanisms between the gut microbiota and the host will help develop more effective intervention strategies. Lastly, environmental factors such as the use of antibiotics can also have a significant impact on the gut microbiota. The misuse of antibiotics can lead to dysbiosis of the gut microbiota and an increase in the proportion of antibiotic-resistant strains [90]. Therefore, the use of antibiotics should be cautious, and their long-term impact on the microbiota should be considered. In summary, reshaping the gut microbiota has broad prospects in the treatment of intestinal damage caused by addictive substance use disorder. Future research should continue to explore the regulatory mechanisms of the gut microbiota and develop effective intervention strategies to improve the intestinal health and overall health status of individuals with addictive substance use disorder.

## Data Availability

No datasets were generated or analysed during the current study.

## Abbreviations

IBD: Inflammatory bowel disease
IBS: Irritable bowel syndrome
METH: Methamphetamine
OR: Opioid receptor
TJs: Tight junctions
AJs: Adherens junctions
GALT: Gut-associated lymphoid tissue
CNS: Central nervous system
BBB: Blood–brain barrier
FMT: Fecal microbiota transplantation
SCFA: Short chain fatty acids
